# High Estimated Glomerular Filtration Rate Is Associated With Worse Cognitive Performance in the Hypertensive Population: Results From the China H-Type Hypertension Registry Study

**DOI:** 10.3389/fnagi.2021.706928

**Published:** 2022-02-17

**Authors:** Junpei Li, Shichao Yu, Ziheng Tan, Yun Yu, Linfei Luo, Wei Zhou, Linjuan Zhu, Tao Wang, Tianyu Cao, Jianglong Tu, Huihui Bao, Xiao Huang, Xiaoshu Cheng

**Affiliations:** ^1^Department of Cardiovascular, Nanchang University Second Affiliated Hospital, Nanchang, China; ^2^Qinghua Health Center, Nanyang, China; ^3^Center for Prevention and Treatment of Cardiovascular Diseases, Nanchang University Second Affiliated Hospital, Nanchang, China; ^4^Department of Biological Anthropology, University of California, Santa Barbara, Santa Barbara, CA, United States; ^5^Department of Neurology, The Second Affiliated Hospital of Nanchang University, Nanchang, China

**Keywords:** estimated glomerular filtration rate (eGFR), cognitive function, mini-mental state examination (MMSE), hypertension, U-shaped

## Abstract

**Background:**

Increasing studies have focused on the predictive value of high estimated glomerular filtration rate (eGFR) on cardiovascular diseases and mortality; however, the association between high eGFR with cognitive function is still not established. Thus, this study aimed to determine the co-relationship between high eGFR and cognitive performance in the hypertensive population.

**Methods:**

We conducted a baseline cross-sectional study using data from the China H-type Hypertension Registry study. Mini-Mental State Examination (MMSE) assessment was performed to evaluate the cognitive function scale, and serum creatinine was collected to estimate eGFR level. Different MMSE cutoff values were applied in participants with the various educational background to define dementia: <24 in participants with secondary school and above education setting, <20 in those with primary school, and <17 in illiterate participants.

**Results:**

A total of 9,527 hypertensive adults with mean age 63.7 ± 9.8 years and 67% female gender were analyzed. The eGFR cutoff value of 71.52 ml/min/1.73 m^2^ was found after adjusting for potential covariates in a threshold effect analysis. The MMSE increased significantly with the increment of eGFR (β, 0.27; 95% CI: 0.12–0.41) in participants with eGFR < 71.52 ml/min/1.73 m^2^ and decreased (β, −0.28; 95% CI: −0.39 to −0.17) in participants with eGFR ≥ 71.52 ml/min/1.73 m^2^. Individuals with eGFR ≥ 85 ml/min/1.73 m^2^ have an elevated risk of cognitive impairment than those with eGFR of 65–75 ml/min/1.73 m^2^. Subgroup analysis showed that a greater reduction degree of MMSE was observed in female individuals and those who had body mass index (BMI) ≥ 24 kg/m^2^ among participants with eGFR ≥ 71.52 ml/min/1.73 m^2^.

**Conclusion:**

Our findings observed an inverted U-shaped relationship between eGFR and cognitive function. Both the low and high levels of eGFR were independently associated with worse cognitive assessment in the hypertensive population.

## Introduction

Chronic kidney disease (CKD) and dementia are major global health concerns, especially in China, which suffered the most from the dementia burden (Collaborators, 2019; Collaboration, 2020). Previous studies have observed that estimated glomerular filtration rate (eGFR) reduction is significantly associated with dementia (Jia et al., 2020). Data from the Alzheimer’s Disease Neuroimaging Initiative database showed that hippocampal atrophy, which is critical for cognitive function, in subjects with eGFR of ≥ 90 ml/min/1.73 m^2^ progressed at half the rate of those with eGFR of 75–90 ml/min/1.73 m^2^ group (An et al., 2017). Auriel et al. (2016) reported that renal function impairment independently predicts 2 years of cognitive decline among survivors who had mild-to-moderate ischemic stroke/transient ischemic attack and without a history of dementia. While low eGFR was identified as an independent risk factor for cognitive impairment, the clinical importance of high eGFR was not well-established and has been frequently overlooked. High eGFR was traditionally used as a surrogate for the sum of all-nephron hyperfiltration (Denic et al., 2017). Increasing studies have suggested that high eGFR is independently associated with cardiovascular and all-cause mortality (van der Sande et al., 2017), coronary artery calcification (Choi et al., 2015), and ventricular hypertrophy (Schmieder et al., 1990; Eriksen et al., 2014).

Hypertension is the most important modifiable risk factor for a large number of diseases, including dementia and CKD, which influence more than 1.3 billion people worldwide (Mills et al., 2020). The PEACE Million Persons Project enrolled about 1.7 million adults aged 35–75 years in China and reported that nearly half (44.7%) of the population had hypertension (Lu et al., 2017). Previous studies suggested that glomerular hyperfiltration may be involved in hypertension-induced target organ damage, especially renal impairment, which was usually accompanied by the activation of the intrarenal renin-angiotensin system and the glomerular changes due to afferent and efferent arteriolar resistance (Schmieder et al., 1990; Fan et al., 2020). However, less has been known about the relationship between high eGFR and cognitive function in a hypertensive population.

In this case, the purpose of this study was to determine the relationship between high eGFR and cognitive function among Chinese hypertensive adults.

## Materials and Methods

### Participant Characteristics

This was a cross-sectional study design. All participants were enrolled from the China H-type Hypertension Registry Study (registration number: ChiCTR1800017274). The study was conducted under the Declaration of Helsinki (World Medical, 2013), and the Ethics Committee of the Institute of Biomedicine, Anhui Medical University approved the protocol. All participants provided written informed consent.

A detailed description of the China H-type Hypertension Registry Study has been described previously (Li et al., 2020). Briefly, the China H-type Hypertension Registry Study was a real-world, observational study conducted from March 2018. Eligible participants were male and female individuals aged 18 years and older and had hypertension, defined as (1) those with resting systolic blood pressure (SBP) ≥ 140 mmHg or diastolic blood pressure (DBP) ≥ 90 mmHg at both the screening and enrollment visit, or (2) those who were on antihypertensive medication.

A total of 14,234 hypertensive participants enrolled in the China H-type Hypertensive Registry in Wuyuan County, China. After excluding participants with incomplete MMSE questionnaires, eGFR data missing (*n* = 3,945), and people with self-reported stroke (*n* = 762), 9,527 participants were included in the final analysis (Supplementary Figure 1).

### Clinical Data Collection

Standardized questionnaires were used to collect demographic information and lifestyle of participants (e.g., age, sex, education, smoking status, and alcohol consumption), as well as self-reported medical and medication history (e.g., stroke, diabetes mellitus, coronary heart disease, antihypertensive drugs, and glucose-lowering drugs). The value of height, weight, and sitting blood pressure was measured by well-trained staff based on the standard procedures. Body mass index (BMI) was calculated as the weight (kg) divided by the squared height (m^2^). Diabetes mellitus was defined as self-reported or physician-diagnosed by the use of glucose-lowering drugs or as a fasting glucose concentration ≥ of 7.0 mmol/L.

Overnight fasting venous blood samples were obtained from each participant at enrollment. Serum creatinine, homocysteine, fasting lipids [including triglycerides, total cholesterol, high-density lipoprotein (HDL)], and fasting glucose were measured using automatic clinical analyzers (Beckman Coulter) at the core laboratory of the National Clinical Research Center for Kidney Disease, Guangzhou, China.

### Estimation of Renal Function and Cognitive Assessments

Our study was conducted in the hypertensive population, mainly with normal and mild renal impairment. Renal function was estimated as eGFR using the Chronic Kidney Disease Epidemiology Collaboration (CKD-EPI) equation (Levey et al., 2009). The CKD-EPI equation more accurately classified individuals based on the risk of mortality and end-stage renal disease (ESRD) than did the Modification of Diet in Renal Disease (MDRD) formula (Matsushita et al., 2012).

Participants were evaluated for cognitive function using the Chinese version of the Mini-Mental State Examination (MMSE) (Li et al., 2016). The MMSE is a widely used test for cognitive function and includes a broad set of cognitive domains, such as orientation, immediate recall, short-term verbal memory, attention and calculation, and language and visuospatial construction. The participants would receive a maximum score of 30 points, representing the highest cognitive function level, by responding to all questions correctly. The screening cutoff value < 24 in participants with secondary school and above education setting (≥ 7 years of education), < 20 in those with primary school (1–6 years of education), and < 17 in illiterate participants were used to assess the occurrence of dementia.

### Statistical Analysis

The clinical cutoff value of eGFR stratified the population characteristics description. Descriptive continuous variables were expressed as mean ± SD, and categorical variables were reported as frequencies and percentages. Chi-square tests were used to compare characteristic differences in categorical variables, while continuous variables were compared between the groups using ANOVA. The dose-response relationship between eGFR and MMSE score was estimated using generalized additive regression model and smoothing curve (penalized spline method) with adjustment for sex, age, education, BMI, SBP, DBP, coronary heart disease, diabetes mellitus, smoking status, alcohol consumption, homocysteine, total cholesterol, triglycerides, HDL, and antihypertensive drugs. Given the definition of mild cognitive impairment (MCI), we had not performed adjustments for education when further exploring the association of eGFR values with dementia. Variables known as traditional or suspected risk factors for kidney function or those that showed significant differences across cognitive impairment were selected as covariables in the regression analyses model. The eGFR is a well-validated formula that takes serum creatinine, age, BMI, and sex into account. To avoid the co-linearity of these variables, collinearity was tested using the Variance Inflation Factor (VIF) criterion. Ultimately, all VIF coefficients were < 5. (Of these, VIF_*age*_: 2.1, VIF_*BMI*_: 1.3, VIF_*sex*_: 2.5, and VIF_*eGFR*_: 1.5.) Hence, no collinearity was present among all independent variables. If the non-linear association was detected in the regression analyses model, we further applied a two piecewise regression model to examine the threshold value effect of eGFR levels and MMSE. The turning point was determined using the likelihood ratio test and bootstrap resampling method. Then, eGFR levels were divided into eight groups at 10 ml/min/1.73 m^2^ intervals, and the interval containing turning point was used as reference groups to validate the non-linear association of eGFR with MMSE and dementia. As further exploratory analyses, possible modifications of varied subgroups on the association of eGFR levels and MMSE scores were evaluated by stratified analyses and interaction testing.

Data were analyzed using the Empower (R^1^; X&Y Solutions, Inc., Boston, MA, United States) and the statistical package R (The R Foundation^2^). A two-tailed *p* < 0.05 was considered statistically significant.

## Results

### Participant Characteristics

Overall, 9,527 participants with completed MMSE questionnaire eGFR data were included in this study. The mean age was 63.7 ± 9.8 years and 67% (*n* = 4,755) were women. The mean MMSE and eGFR were 22.1 ± 6.4 and 86.3 ± 19.6 ml/min/1.73 m^2^, respectively. Participants with higher eGFR (≥ 90 ml/min/1.73 m^2^) were more likely to be younger, female, and have higher BMI, DBP, MMSE score (including MMSE subscores), and percentages of current drinking, but lower levels of homocysteine, fasting glucose, SBP, percentages of current smoking, illiteracy, diabetes mellitus, coronary heart disease, dementia, and the use of antihypertensive drugs (Table 1). Supplementary Table 1 presents the usage of antihypertensive drugs in detail. In brief, calcium channel blocker (CCB) was the most commonly used antihypertensive drug in this study population, approximately 1 in 10 people used angiotensin-converting enzyme inhibitor (ACEI).

**TABLE 1:** Characteristics of subjects stratified by the clinical cutoff value of eGFR.

Characteristics	Total	eGFR, ml/min/1.73 m^2^			*P*-value
		<60	≥ 60, <90	≥90	
N	9,527	1,015	3,496	5,016	
Age, years	63.7 ± 9.8	70.8 ± 9.1	67.5 ± 9.0	59.5 ± 8.5	<0.001
Female, N (%)	4,955 (52.0)	462 (45.5)	1,672 (47.8)	2,821 (56.2)	<0.001
BMI, kg/m^2^	23.6 ± 3.5	22.9 ± 3.6	23.3 ± 3.5	23.9 ± 3.5	<0.001
SBP, mmHg	147.2 ± 17.5	149.2 ± 20.9	148.0 ± 18.3	146.3 ± 16.1	<0.001
DBP, mmHg	89.0 ± 10.8	85.5 ± 12.1	87.8 ± 11.0	90.6 ± 10.1	<0.001
**Laboratory results**					
Homocysteine, μmol/L	17.9 ± 11.1	25.6 ± 15.6	19.2 ± 11.6	15.3 ± 8.4	<0.001
Fasting glucose, mmol/L	6.2 ± 1.6	6.3 ± 1.7	6.2 ± 1.6	6.2 ± 1.5	0.009
Total cholesterol, mmol/L	5.1 ± 1.1	5.1 ± 1.2	5.1 ± 1.1	5.1 ± 1.1	0.065
Triglyceride, mmol/L	1.8 ± 1.3	1.8 ± 1.3	1.8 ± 1.2	1.9 ± 1.4	<0.001
HDL, mmol/L	1.5 ± 0.4	1.5 ± 0.4	1.5 ± 0.4	1.5 ± 0.4	0.004
eGFR, ml/min/1.73 m^2^	86.3 ± 19.6	44.9 ± 13.1	78.3 ± 8.6	100.3 ± 7.3	<0.001
**Cognitive Function**					
MMSE	22.1 ± 6.4	20.6 ± 6.8	21.9 ± 6.4	22.5 ± 6.3	<0.001
Orientation	8.1 ± 2.3	7.8 ± 2.5	8.0 ± 2.3	8.2 ± 2.2	<0.001
Immediate recall	2.3 ± 1.0	2.1 ± 1.2	2.3 ± 1.1	2.4 ± 1.0	<0.001
Calculation and attention	2.7 ± 2.0	2.4 ± 2.0	2.7 ± 2.0	2.8 ± 1.9	<0.001
Short-term verbal memory	1.9 ± 1.2	1.6 ± 1.3	1.8 ± 1.2	2.0 ± 1.2	<0.001
Language and visual-spatial skills	7.0 ± 1.7	6.7 ± 1.8	7.0 ± 1.7	7.1 ± 1.7	<0.001
Smoking status, N(%)					<0.001
Never	5,434 (57.0)	510 (50.2)	1,823 (52.1)	3,101 (61.8)	
Former	1,593 (16.7)	237 (23.3)	701 (20.1)	655 (13.1)	
Current	2,500 (26.2)	268 (26.4)	972 (27.8)	1,260 (25.1)	
Alcohol consumption, N(%)					<0.001
Never	6,432 (67.5)	703 (69.3)	2,327 (66.6)	3,402 (67.8)	
Former	963 (10.1)	157 (15.5)	386 (11.0)	420 (8.4)	
Current	2,132 (22.4)	155 (15.3)	783 (22.4)	1,194 (23.8)	
Education, N(%)					<0.001
Illiteracy	3,478 (36.5)	405 (39.9)	1,309 (37.4)	1,764 (35.2)	
Primary school	4,024 (42.2)	438 (43.2)	1,466 (41.9)	2,120 (42.3)	
Secondary school and above	2,025 (21.3)	172 (16.9)	721 (20.6)	1,132 (22.6)	
Diabetes mellitus, N(%)	1,727 (18.1)	246 (24.2)	611 (17.5)	870 (17.3)	<0.001
Coronary heart disease, N(%)	533 (5.6)	76 (7.5)	254 (7.3)	203 (4.0)	<0.001
Mild cognitive impairment, N(%)	2,541 (26.7)	344 (33.9)	993 (28.4)	1,204 (24.0)	<0.001
Antihypertensive drugs, N(%)	5,798 (60.9)	753 (74.2)	2,270 (64.9)	2,775 (55.3)	<0.001

*For continuous variables, values are presented as mean ± SD; for categorical variables, values are presented as N(%).*

*BMI, body mass index; SBP, systolic blood pressure; DBP, diastolic blood pressure; HDL, high-density lipoprotein; eGFR, estimated glomerular filtration rate; MMSE, Mini-Mental State Examination.*

### Association Between Estimated Glomerular Filtration Rate and Cognitive Function

**FIGURE 1:**
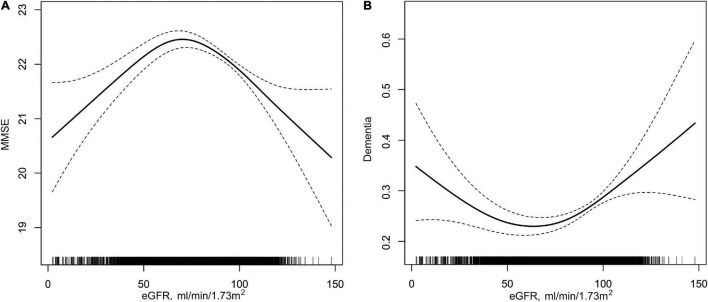
Association of eGFR with MMSE and dementia in hypertensive population. **(A)** eGFR with MMSE; **(B)** eGFR with dementia. Solid lines in **(A,B)** represent β value and OR value, respectively; two dotted lines indicate 95% CIs. All analyses were adjusted for sex, age, body mass index, systolic blood pressure, diastolic blood pressure, education (except in **B**), coronary heart disease, diabetes mellitus, smoking status, alcohol consumption, homocysteine, total cholesterol, triglycerides, high-density lipoprotein, and antihypertensive drugs.

**FIGURE 2:**
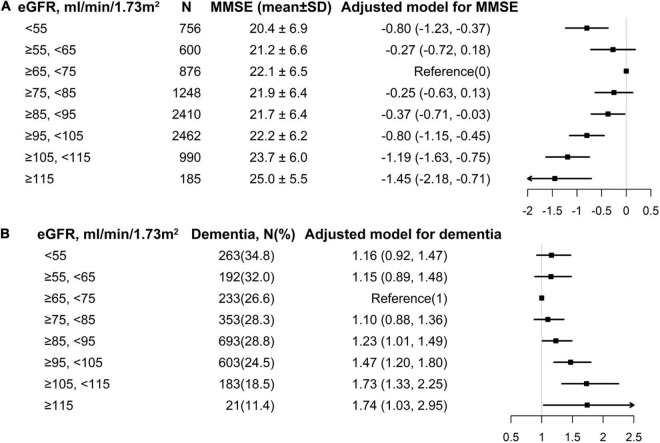
Multivariate logistic regression of eGFR with MMSE and dementia among individuals divided into 10 ml/min/1.73 m^2^ intervals of eGFR levels. **(A)** eGFR with MMSE; **(B)** eGFR with dementia. Each black square represents the effect size of the study together with the 95% CI. **(A)** eGFR with MMSE; **(B)** eGFR with dementia. All analyses were adjusted for sex, age, body mass index, systolic blood pressure, diastolic blood pressure, education (except in Figure 1B), coronary heart disease, diabetes mellitus, smoking status, alcohol consumption, homocysteine, total cholesterol, triglycerides, high-density lipoprotein, and antihypertensive drugs.

Overall, a non-linear association of eGFR with MMSE score and dementia had been detected in the smoothing curve. Both the low and high levels of eGFR were associated with worse cognitive performance, while participants with the intermediate level of eGFR had better performance in cognitive assessment (Figure 1). A turning point of 71.52 ml/min/1.73 m^2^ yielded the best fitting model in two piecewise regressions, after fully adjusting for sex, age, BMI, SBP, DBP, education, coronary heart disease, diabetes mellitus, smoking status, alcohol consumption, homocysteine, total cholesterol, triglycerides, HDL, and antihypertensive drugs. Further adjusting the types of antihypertensive drugs, including CCBs, ACEIs, angiotensin-receptor blockers, β-blockers, diuretic, and other antihypertensive drugs, the above analysis was repeated to minimize the confounding factors of antihypertensive drugs type on eGFR (Supplementary Figures 2, 3). Notably, the result remains stable with the previous analysis. The MMSE subscores show similar inversed U-shaped trends with eGFR levels (Supplementary Figure 4). The MMSE score increased significantly with the increment of eGFR (per 10 ml/min/1.73 m^2^ increase: β, 0.27; 95% CI: 0.12–0.41) in participants with eGFR < 71.52 ml/min/1.73 m^2^ and decreased (per 10 ml/min/1.73 m^2^ increase: β, −0.28; 95% CI: −0.39 to −0.17) in participants with eGFR ≥ 71.52 ml/min/1.73 m^2^ (Table 2). Supplementary Figure 5 shows the number and mean MMSE score of individuals classified according to eGFR and age. The eGFR of 65–85 ml/min/1.73 m^2^ was at the peak of the mean MMSE score in all ages group within all age bands and, thus, better cognitive performance. Consistently, when eGFR was assessed as eight groups at 10 ml/min/1.73 m^2^ interval, compared with those with eGFR ≥ 65 and < 75 ml/min/1.73 m^2^, participants with both eGFR < 65 and ≥ 75 ml/min/1.73 m^2^ were inversely associated with the performance of MMSE assessment. Individuals with eGFR ≥ 115 ml/min/1.73 m^2^ had a 1.45-fold increased risk of cognitive impairment than those with eGFR of 65–75 ml/min/1.73 m^2^ (Figure 2A). Consistently, Figure 2B presents the relative odds of dementia. Compared to participants with eGFR ≥ 65 and < 75 ml/min/1.73 m^2^, the adjusted odds ratio (OR) for participants with eGFR ≥ 105 and <115 ml/min/1.73 m^2^ and participants with eGFR ≥ 115 ml/min/1.73 m^2^ were 1.73 (95% CI: 1.33–2.25) and 1.74 (95% CI: 1.03–2.95), respectively.

**TABLE 2:** Threshold effect analyses of the relationship between eGFR and MMSE using two piecewise regression models.

eGFR, ml/min/1.73 m^2^	Subjects	MMSE Mean ± SD	Crude model β (95% CI)	*P*-value	Adjusted model β (95% CI)	*P*-value
Per 10 ml/min/1.73 m^2^ increase	9,527	22.1 ± 6.4	0.38 (0.32, 0.45)	<0.001	−0.04 (−0.10, 0.01)	0.122
**Turning point**						
<71.52	1,919	21.2 ± 6.7	0.50 (0.30, 0.71)	<0.001	0.27 (0.12, 0.41)	<0.001
≥ 71.52	7,608	22.3 ± 6.3	0.59 (0.46, 0.72)	<0.001	−0.28 (−0.39, −0.17)	<0.001
*P* for log-likelihood ratio test				0.461		<0.001

*eGFR, estimated glomerular filtration rate; MMSE, Mini-Mental State Examination.*

*Adjusted for: sex, age, body mass index, systolic blood pressure, diastolic blood pressure, education, coronary heart disease, diabetes mellitus, smoking status, alcohol consumption, homocysteine, total cholesterol, triglycerides, high-density lipoprotein, and antihypertensive drugs.*

### Subgroups Analyses

**FIGURE 3:**
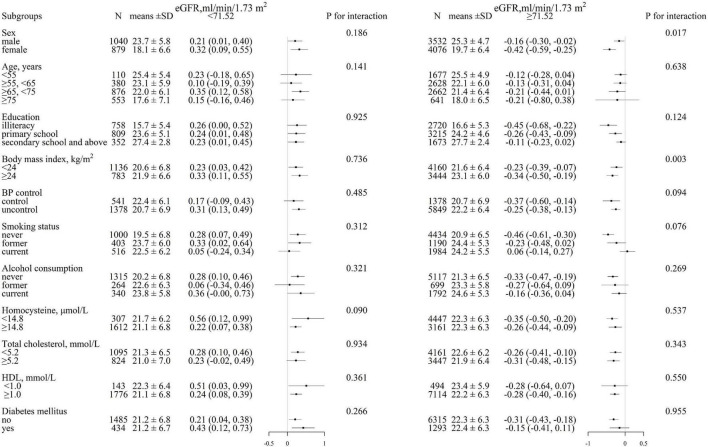
Stratified analysis for MMSE and eGFR in various subgroups divided by 71.52 ml/min/1.73 m^2^. (eGFR < 71.52 ml/min/1.73 m^2^, eGFR ≥ 71.52 ml/min/1.73 m^2^)*. Each black square represents the effect size of the study together with the 95% CI. Each subgroup analysis adjusted for sex, age, body mass index, systolic blood pressure, diastolic blood pressure, education, coronary heart disease, diabetes mellitus, smoking status, alcohol consumption, homocysteine, total cholesterol, triglycerides, high-density lipoprotein, and antihypertensive drugs, except for the stratification variable.

We performed an exploratory subgroup analysis to further assess the association between eGFR levels and cognitive function in two groups of participants divided according to the turning point of eGFR (71.52 ml/min/1.73 m^2^) (Figure 3). The significant interactions were found in the subgroup of sex (*p* for interaction = 0.017) and BMI (*p* for interaction = 0.003) among participants with eGFR ≥ 71.52 ml/min/1.73 m^2^, a greater degree of MMSE decrease was observed in female individuals and those with BMI ≥ 24 kg/m^2^, whereas in the other subgroups, including age (<55 vs. 55– < 65 vs. 65– < 75 vs. ≥ 75 years), education (illiteracy vs. primary school vs. secondary school and above), blood pressure control (yes vs. no), smoking status (never vs. former vs. current), alcohol consumption (never vs. former vs. current), homocysteine (< 15 vs. ≥ 15 μmol/L), total cholesterol (< 5.2 vs. ≥ 5.2 mmol/L), HDL (< 1.0 vs. ≥ 1.0 mmol/L), and diabetes mellitus (yes vs. no), no differences have been observed (all *p* for interaction > 0.05).

## Discussion

This study explored the association between a high level of eGFR and cognitive impairment, meanwhile reconfirming the predictive value of low eGFR to cognitive performance. The MMSE score was lowest at a turning point of eGFR (71.52 ml/min/1.73 m^2^), which was revealed by a threshold effect analysis, and increased at both the lower and higher levels of eGFR. In addition, for those with eGFR ≥ 71.52 ml/min/1.73 m^2^, a stronger positive association between eGFR levels and cognitive impairment was found in participants who were female and had high BMI (≥ 24 kg/m^2^).

### Low Estimated Glomerular Filtration Rate and Cognitive Function

The association between impaired renal function and cognitive function had been investigated by previous studies. The Cardiovascular Health Study and the Rush Memory and Aging Project reported that cognitive function decline was associated with decreasing eGFR in the elderly population (Seliger et al., 2004; Buchman et al., 2009). The aforementioned relationship also significantly present in stroke/transient ischemic attack survivors, Auriel et al. (2016) and Ben Assayag et al. (2017) found that impaired renal function (defined by estimated creatinine clearance < 60 ml/min) was associated with hippocampal volume atrophy and poststroke cognitive function decline (Auriel et al., 2016; Ben Assayag et al., 2017). This study observed that lower eGFR was associated with worse cognitive performance in a Chinese population after adjusting for potential covariates, which was in agreement with previous studies. However, the mechanism behind such a co-relationship has remained unknown. Kidney-brain axis theories contribute several pathologies, including vascular endothelial injury and chronic neuroinflammation due to the accumulation of renal-related toxins in serum, which further leads to a decrease in cognitive function (Bugnicourt et al., 2013; Shi et al., 2018).

### High Estimated Glomerular Filtration Rate and Cognitive Function

Recently, the study about the relationship of high eGFR with adverse events had been given importance. Studies that examined the association between high eGFR levels and adverse outcomes were mainly conducted on individuals with diabetes mellitus (Silveiro et al., 1996; Amin et al., 2005; Groop et al., 2009). The recognized mechanisms attribute higher eGFR levels to incipient hyperfiltration and developing microalbuminuria in the early development stages for diabetes mellitus (Vedel et al., 1996). A cross-sectional study by Choi et al. (2015) revealed that high eGFR was associated with coronary artery calcification among middle-aged men without CKD when compared with eGFR of 75–89 ml/min/1.73 m^2^. A significant correlation was also observed in subgroups analysis among individuals who had no hypertension and diabetes mellitus (Choi et al., 2015). Glomerular hyperfiltration associated with increased cardiovascular risk was also found in healthy middle-aged individuals from a prospective population-based cohort study (Dupuis et al., 2020). Putaala et al. (2011) carried out a study for 958 young and middle-aged Finnish patients with a history of first-ever ischemic stroke and found that high eGFR of > 120 ml/min/1.73 m^2^ independently predicted long-term all-cause mortality. The studies mentioned above mainly focused on the relationship between high eGFR and cardiovascular disease and death, but the association of high eGFR with cognitive impairment was not well-established. Weiner et al. (2017) reported a threshold eGFR effect in the SPRINT-MIND study that participants with eGFR > 90 ml/min/1.73 m^2^ had slightly poorer performance on global cognitive function and executive function domains than those with eGFR of 75–90 ml/min/1.73 m^2^. Consistently, our study found that among hypertensive adults, individuals with eGFR ≥ 85 ml/min/1.73 m^2^ have an elevated risk of cognitive impairment than those with eGFR of 65–75 ml/min/1.73 m^2^. Individuals with eGFR of ≥ 115 ml/min/1.73 m^2^ had a 1.74-fold increased risk of dementia than those with eGFR of 65–75 ml/min/1.73 m^2^. Results between diabetic and non-diabetic populations were congruent according to the subgroup analysis (p for interaction > 0.05).

The mechanisms of the association between high eGFR and worse cognitive function were not fully understood. Increased arterial stiffness may play a key role in the association between high eGFR and cognitive decline among the hypertensive population. It was well-known that increased aortic stiffness was independently associated with worse cognitive performance (Sha et al., 2018; Rensma et al., 2020). Reboldi et al. (2018) reported that compared with normal eGFR participants, those with high eGFR had a wider ambulatory pulse pressure interval and abnormal nighttime blood pressure rhythm, which suggested increased large arterial stiffness and sympathetic nervous system activity (Dart and Kingwell, 2001). Lin et al. (2017) also found in the Chinese population that high eGFR was associated with arterial stiffness manifested by elevated brachial-ankle pulse wave velocity and pulse pressure. Multiple biological pathways coexist in both increased arterial stiffness and glomerular hyperfiltration. Moreover, both hyperfiltration and arterial stiffness can be present at the early phase of hypertension, which also, in turn, induce further stiffening of the arteries and increase glomerular filtration pressure (Harrap et al., 2000; Safar, 2018; Safar et al., 2018). The renin-angiotensin-aldosterone and sympathetic nervous system activity were implicated as contributing pathways to arterial stiffness and glomerular hyperfiltration in hypertension by stimulating collagen accumulation (Ratto et al., 2006; Helal et al., 2012; Fan et al., 2020).

Another possible explanation for the association between high eGFR values with cognitive impairment might be obesity, a manifestation of high BMI (Studenski et al., 2014). According to the population characteristics in this study, it was shown that individuals with high eGFR were prone to have higher BMI than those with low eGFR. In animal research, the induction of the obese models caused increased renin-angiotensin system activity, GFR, and renal plasma flow (Henegar et al., 2001). Population-based studies also reported that overweight and obesity were associated with increased GFR, effective renal plasma flow, and filtration fraction (Wuerzner et al., 2010; Turer et al., 2019). High BMI was considered a risk factor for a wide range of health conditions, including various cardiovascular complications and insulin resistance, which all contribute to the increased risk of dementia (De Felice and Ferreira, 2014). It had been widely reported that various obesity anthropometric indicators (e.g., BMI and waist circumference) were inversely associated with cognitive function (Cournot et al., 2006; Cheke et al., 2017; Dye et al., 2017). The Swedish Twin Registry showed that high BMI at midlife independently increases the risk of dementia, Alzheimer’s disease, and vascular dementia in later life (Xu et al., 2011). Although BMI, potential covariates, was adjusted in our regression analysis, a stronger association between high eGFR (≥ 71.52 ml/min/1.73 m^2^) and MMSE in women and with a BMI of ≥ 24 kg/m^2^ was still found in subgroup analysis. These observations highlight that overweight/obesity, especially in women, may place an extra burden on nephrons, contribute to glomerular hyperfiltration, and aggravate cognitive decline over time.

Weiner et al. (2017) proposed that high eGFR does not reflect an accurate hyperfiltration but rather attributed high creatinine-based eGFR to cachexia with low serum creatinine concentration. If this hypothesis was correct, the previous results on high GFR and adverse outcomes should be interpreted by low creatinine concentrations. Eriksen et al. (2014) reported that measured GFR by iohexol clearance was manifested in the association between high GFR and subclinical cardiovascular disease, including carotid atherosclerosis and left ventricular hypertrophy, which implies that the interpretation of cachexia with low serum creatinine concentration has remained controversial.

### Limitations

Several potential concerns or limitations are worth mentioning. First, current findings should be considered preliminary given the study limitations of cross-sectional design, further longitudinal studies with follow-up for change of cognitive function are needed. In addition, whether the intervention to improve the high filtration state can alleviate cognitive impairment needs to be further verified by more rigorously designed randomized controlled trials. Second, the eGFR estimated by the CKD-EPI equation in this study may not perfectly represent real GFR. Although the CKD-EPI equation has been well-validated, the accurate measured GFR is still needed in future studies to establish the relationship between high GFR and cognition. Third, as with all observational studies, although we adjusted for multiple potential confounders, we cannot exclude the possibility of residual confounding, including, but not limited to, ApoE genotyping, social factors, brain atrophy, and cerebral small vessel disease. Also, due to the lack of data on urinalysis (i.e., proteinuria and urine creatinine), this study was unable to validate the role of proteinuria on the relationship of high eGFR with cognition. However, Tonelli et al. (2011) found that higher eGFR was associated with increased risk of adverse outcomes, especially with concomitant proteinuria. Consequently, we speculate that concomitant proteinuria may also well play such a role in the relationship between high eGFR and cognition. Fourth, this study participants have only assessed the cognitive function by the MMSE questionnaire, which has limited sensitivity and specificity (range from 87.6 to 94.3% and 80.8 to 94.3% in elderly Chinese, respectively, Li et al., 2016) in screening dementia, while the collaborative evaluation of cognitive function by multiple questionnaires may improve the effectiveness of the assessment. Finally, our participants were hypertensive patients, the glomerular hyperfiltration in essential hypertension suggested early target organ damage (Schmieder et al., 1990), which may also contribute to worse cognitive performance. Thus, the discovered results may not be generalized to other populations. Given that cardiovascular events were closely related to cognitive function, we believe our findings warranted to be validated in the studies of other populations.

## Conclusion

Both the low and high levels of eGFR were independently associated with worse cognitive function among the Chinese hypertensive population, especially in the female gender and in those with BMI ≥ 24 kg/m^2^. Individuals with eGFR ≥ 85 ml/min/1.73 m^2^ have an elevated risk of cognitive impairment than those with eGFR of 65–75 ml/min/1.73 m^2^. Results from this study implied that special attention should be paid to hypertensive patients with high eGFR, which could provide an easily identifiable marker of worse cognitive performance.

## Data Availability Statement

The datasets presented in this article are not readily available because data access is obtained according to individual contributions to the study. Requests to access the datasets should be directed to the corresponding authors.

## Ethics Statement

The studies involving human participants were reviewed and approved by the Ethics Committee of the Institute of Biomedicine, Anhui Medical University. The patients/participants provided their written informed consent to participate in this study.

## Author Contributions

JL, HB, XH, and XC came up with ideas and designed the study. JL, SY, ZT, YY, LL, WZ, LZ, TW, HB, XH, XC, and all staff of the China H-type Hypertension Registry Study collected data. JL, WZ, HB, XH, and XC performed the statistical analysis. JL, XH, and XC wrote the first draft. TC contributed to English language editing. JL, JT, HB, XH, and XC reviewed and revised the article. All authors approved the final version.

## Conflict of Interest

The authors declare that the research was conducted in the absence of any commercial or financial relationships that could be construed as a potential conflict of interest.

## Publisher’s Note

All claims expressed in this article are solely those of the authors and do not necessarily represent those of their affiliated organizations, or those of the publisher, the editors and the reviewers. Any product that may be evaluated in this article, or claim that may be made by its manufacturer, is not guaranteed or endorsed by the publisher.

## Footnotes

1
www.empowerstats.com

2
http://www.R-project.org
