# Supplementary material for: High Estimated Glomerular Filtration Rate Is Associated With Worse Cognitive Performance in the Hypertensive Population: Results From the China H-Type Hypertension Registry Study
Source: Front Aging Neurosci. 2022 Feb 17;13:706928. doi: 10.3389/fnagi.2021.706928 (PMC8893225; doi:10.3389/fnagi.2021.706928)
Supplement: Supplementary file 1 [file Data_Sheet_1.pdf]

High estimated glomerular filtration rate is associated with worse cognitive performance in the hypertensive population: Results from The China H-type Hypertension Registry Study

Supplementary Material

Figure legends

Supplementary Figure 1. Flow diagram of study participants.

Supplementary Figure 2. Association of eGFR with MMSE and dementia in hypertensive population*. (a) eGFR with MMSE; (b) eGFR with dementia. Red solid lines in Supplementary Figure 2a and 2b represent β value and OR value, respectively; Two blue dotted lines indicate 95% confidence intervals. *All analysis were adjusted for sex, age, body mass index, systolic blood pressure, diastolic blood pressure, education (except in figure 1b), coronary heart disease, diabetes mellitus, smoking status, alcohol consumption, homocysteine, total cholesterol, triglycerides, high-density lipoprotein, calcium channel blockers, angiotensin-converting enzyme inhibitors, angiotensin receptor blockers, β-blockers, diuretic and other antihypertensive drugs.

Supplementary Figure 3. Multivariate logistic regression of eGFR with MMSE and dementia among individuals divided into 10 ml/min per 1.73 m^2 intervals of eGFR levels. (a) eGFR with MMSE; (b) eGFR with dementia. Each black square represents the effect size of the study together with the 95% confidence interval. (a) eGFR with MMSE; (b) eGFR with dementia. *All analysis were adjusted for sex, age, body mass index, systolic blood pressure, diastolic blood pressure, education (except in figure 1b), coronary heart disease, diabetes mellitus, smoking status, alcohol consumption, homocysteine, total cholesterol, triglycerides, high-density lipoprotein, calcium channel blockers, angiotensin-converting enzyme inhibitors, angiotensin receptor blockers, β-blockers, diuretic and other antihypertensive drugs.

Supplementary Figure 4. The association of eGFR with orientation (a), immediate recall (b), calculation and attention (c, short-term verbal memory (d) and language and visual-spatial skills (e). The adjusted model adjusts for sex, age, body mass index, systolic blood pressure, diastolic blood pressure, education, coronary heart disease, diabetes mellitus, smoking status, alcohol consumption, homocysteine, total cholesterol, triglycerides, high-density lipoprotein, and anti-hypertensive drugs.
Supplementary Figure 5. The number and the mean MMSE score of individuals classified according to eGFR and age.

Supplementary Table 1. Details of antihypertensive drugs use in study participants.

	eGFR, ml/min/1.73m^2			P-value
	<60	≥60, <90	≥90	
Antihypertensive drugs, N(%)	753 (74.2)	2270 (64.9)	2775 (55.3)	<0.001
Classification, N(%)				
calcium channel blockers	354 (34.9)	1198 (34.3)	1614 (32.2)	0.066
angiotensin-converting enzyme inhibitors	107 (10.5)	299 (8.6)	322 (6.4)	<0.001
angiotensin receptor blockers	24 (2.4)	79 (2.3)	121 (2.4)	0.900
β-blockers	21 (2.1)	51 (1.5)	37 (0.7)	<0.001
diuretic	66 (6.5)	170 (4.9)	166 (3.3)	<0.001
other	181 (17.8)	473 (13.5)	513 (10.2)	<0.001

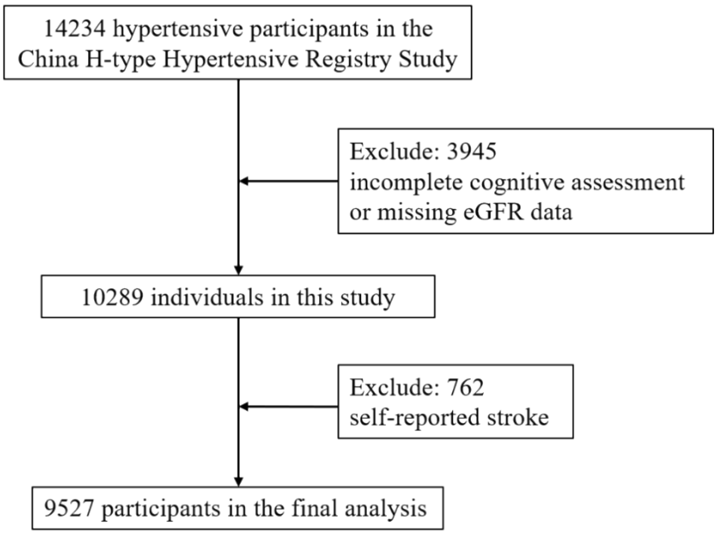

Supplementary Figure 1. Flow diagram of study participants

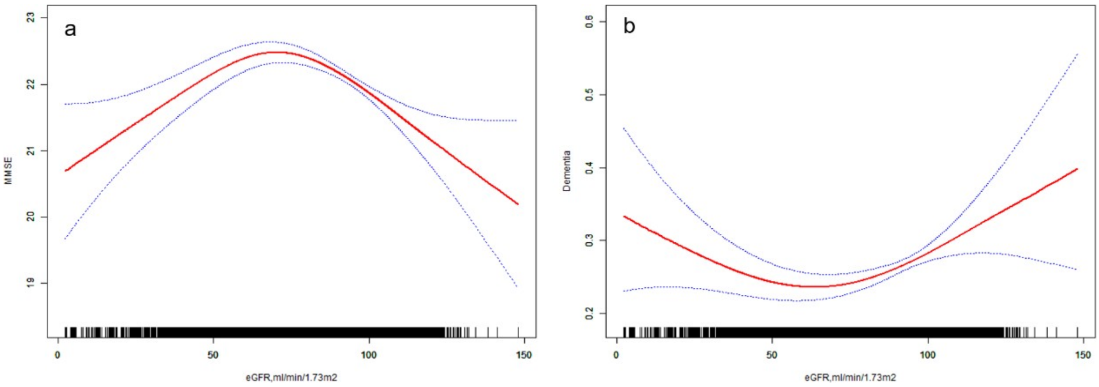

Supplementary Figure 2. Association of eGFR with MMSE and dementia in hypertensive population*. (a) eGFR with MMSE; (b) eGFR with dementia. Red solid lines in Supplementary Figure 2a and 2b represent β value and OR value, respectively; Two blue dotted lines indicate 95% confidence intervals. *All analysis were adjusted for sex, age, body mass index, systolic blood pressure, diastolic blood pressure, education (except in figure 1b), coronary heart disease, diabetes mellitus, smoking status, alcohol consumption, homocysteine, total cholesterol, triglycerides,

high-density lipoprotein, calcium channel blockers, angiotensin-converting enzyme inhibitors, angiotensin receptor blockers, β-blockers, diuretic and other antihypertensive drugs.

a

eGFR, ml/min/1.73m²	N	MMSE (mean±SD)	Adjusted model for MMSE
<55	756	20.4 ± 6.9	-0.80 (-1.23, -0.38)
≥55, <65	600	21.2 ± 6.6	-0.26 (-0.70, 0.19)
≥65, <75	876	22.1 ± 6.5	Reference(0)
≥75, <85	1248	21.9 ± 6.4	-0.28 (-0.65, 0.09)
≥85, <95	2410	21.7 ± 6.4	-0.39 (-0.73, -0.06)
≥95, <105	2462	22.2 ± 6.2	-0.76 (-1.11, -0.42)
≥105, <115	990	23.7 ± 6.0	-1.16 (-1.59, -0.73)
≥115	185	25.0 ± 5.5	-1.43 (-2.15, -0.70)

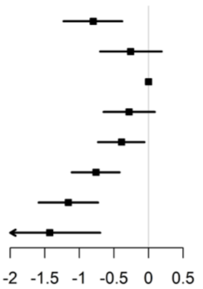

b

eGFR, ml/min/1.73m²	Dementia, N(%)	Adjusted model for dementia
<55	263(34.8)	1.16 (0.91, 1.47)
≥55, <65	192(32.0)	1.15 (0.90, 1.49)
≥65, <75	233(26.6)	Reference(1)
≥75, <85	353(28.3)	1.10 (0.88, 1.36)
≥85, <95	693(28.8)	1.23 (1.02, 1.50)
≥95, <105	603(24.5)	1.48 (1.20, 1.81)
≥105, <115	183(18.5)	1.74 (1.34, 2.27)
≥115	21(11.4)	1.78 (1.05, 3.01)

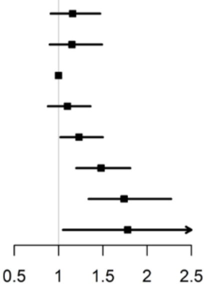

Supplementary Figure 3. Multivariate logistic regression of eGFR with MMSE and dementia among individuals divided into 10 ml/min per 1.73 m^2 intervals of eGFR levels. (a) eGFR with MMSE; (b) eGFR with dementia. Each black square represents the effect size of the study together with the 95% confidence interval. (a) eGFR with MMSE; (b) eGFR with dementia. *All analysis were adjusted for sex, age, body mass index, systolic blood pressure, diastolic blood pressure, education (except in figure 1b), coronary heart disease, diabetes mellitus, smoking status, alcohol consumption, homocysteine, total cholesterol, triglycerides, high-density lipoprotein, calcium channel blockers, angiotensin-converting enzyme inhibitors, angiotensin receptor blockers, β-blockers, diuretic and other antihypertensive drugs.

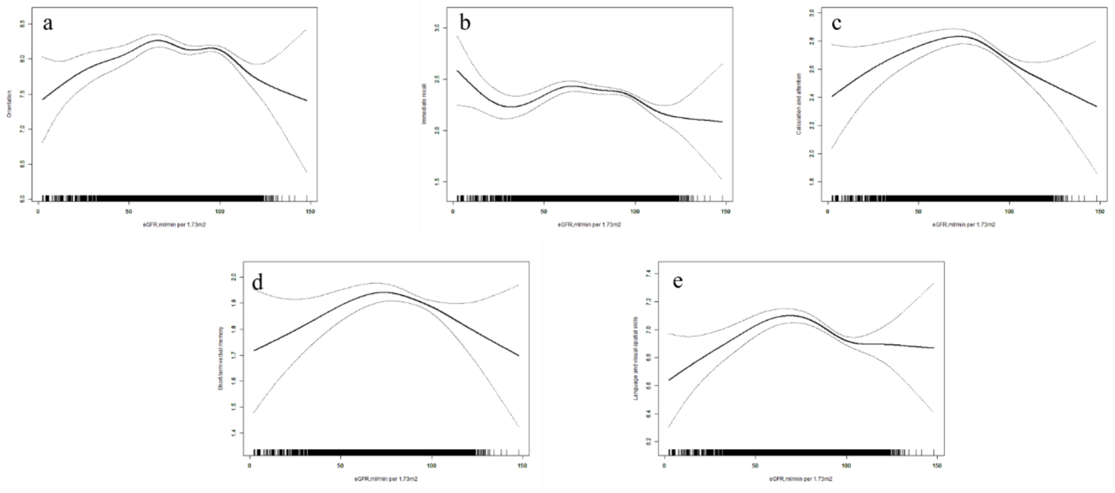

Supplementary Figure 4. The association of eGFR with orientation (a), immediate recall (b), calculation and

attention (c, short-term verbal memory (d) and language and visual-spatial skills (e). The adjusted model adjusts for sex, age, body mass index, systolic blood pressure, diastolic blood pressure, education, coronary heart disease, diabetes mellitus, smoking status, alcohol consumption, homocysteine, total cholesterol, triglycerides, high-density lipoprotein, and anti-hypertensive drugs.

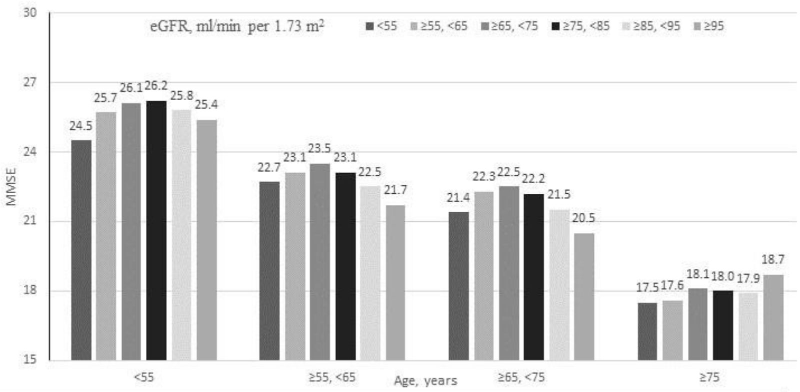

eGFR,ml/min/1.73m^2	N	MMSE	N	MMSE	N	MMSE	N	MMSE
<55	37	24.5 ± 5.4	122	22.7 ± 5.7	332	21.4 ± 6.2	265	17.5 ± 7.3
≥55, <65	30	25.7 ± 5.7	123	23.1 ± 5.8	269	22.3 ± 5.9	178	17.6 ± 6.8
≥65, <75	62	26.1 ± 5.2	221	23.5 ± 5.8	409	22.5 ± 6.1	184	18.1 ± 6.8
≥75, <85	130	26.2 ± 4.2	312	23.1 ± 5.5	524	22.2 ± 6.2	282	18.0 ± 6.6
≥85, <95	206	25.8 ± 4.8	649	22.5 ± 5.9	1301	21.5 ± 6.4	254	17.9 ± 6.4
≥95	1322	25.4 ± 5.0	1581	21.7 ± 6.1	703	20.5 ± 6.5	31	18.7 ± 6.9

Supplementary Figure 5. the number and the mean MMSE score of individuals classified according to eGFR and age.
